# Relative Consistency of Sample Entropy Is Not Preserved in MIX Processes

**DOI:** 10.3390/e22060694

**Published:** 2020-06-21

**Authors:** Sebastian Żurek, Waldemar Grabowski, Klaudia Wojtiuk, Dorota Szewczak, Przemysław Guzik, Jarosław Piskorski

**Affiliations:** 1 Institute of Physics, University of Zielona Gora, 65-417 Zielona Gora, Poland; S.Zurek@if.uz.zgora.pl (S.Ż.); wgrabowski@gmail.com (W.G.); klaudia.wojtiuk.94@gmail.com (K.W.); dladoroty@gmail.com (D.S.); 2 Department of Cardiology-Intensive Therapy, Poznan University of Medical Sciences Poznan, 61-701 Poznan, Poland; pguzik@ptkardio.pl

**Keywords:** time series analysis, sample entropy, relative consistency

## Abstract

Relative consistency is a notion related to entropic parameters, most notably to Approximate Entropy and Sample Entropy. It is a central characteristic assumed for e.g., biomedical and economic time series, since it allows the comparison between different time series at a single value of the threshold parameter *r*. There is no formal proof for this property, yet it is generally accepted that it is true. Relative consistency in both Approximate Entropy and Sample entropy was first tested with the MIX process. In the seminal paper by Richman and Moorman, it was shown that Approximate Entropy lacked the property for cases in which Sample Entropy did not. In the present paper, we show that relative consistency is not preserved for MIX processes if enough noise is added, yet it is preserved for another process for which we define a sum of a sinusoidal and a stochastic element, no matter how much noise is present. The analysis presented in this paper is only possible because of the existence of the very fast NCM algorithm for calculating correlation sums and thus also Sample Entropy.

## 1. Introduction

Relative consistency of Sample Entropy (SampEn) has been assumed in all clinical and economic applications [1,2,3,4]. Indeed, if we say that a one time series is more complex than another on the basis of their value at a certain threshold *r*, we assume this either explicitly or tacitly. It is quite surprising that there are no comprehensive studies on this property of Sample Entropy. The analytic proof of this property would be very hard to derive. In fact, very little theoretical work has been done on these parameters, most of which is limited to the Moorman and Richman paper [4]. We do not know the distribution of Sample Entropy (the *t* distribution assumed in [4] is based on data, not analytical properties), we do not know entropy profiles of most common processes or the influence of noise on these processes. The experimental verification of relative consistency requires calculation of SampEn across many different thresholds for many time series e.g., many RR (i.e., the distance between two consecutive R-vawes in the ECG) intervals time series, acquired with the same equipment (the same sampling rate as well as other external conditions). This is difficult because of the computational burden of this task. In this paper, we overcome this difficulty by using the NCM algorithm [5]. Unlike a formal proof, this procedure would not yield certainty about relative entropy, but it could either corroborate, or decisively refute it.

We believe that the methodology developed in this paper is systematic and applicable to all types of time series studied with the use of Sample Entropy. Furthermore, we provide the software necessary to perform such an analysis quickly and without large hardware investments.

This paper is not the first one to study relative consistency of Sample Entropy as a universal property. Indeed, even the creators of SampEn allow for the possibility of Sample Entropy not holding universally for all time series [4].

In [6], the authors perform an analysis of short time series acquired by recording gait data. The most significant result in the context of the present paper is the finding that SampEn is not relatively consistent at r=0.2 for the time series of step time of length 200—the averaged entropy profiles for short data of young subject cross with the profiles of older subjects. The authors do not provide specific results, i.e., how many curves cross, but nonetheless this is a significant finding for this time series. The cross between entropy profiles in [6] was observed for very short recordings, but this finding was corroborated in longer recordings [7]. The authors find that, for one hour recordings of time series of step time, the relative consistency is lost between overground and treadmill walking recordings at r=0.2 for m=2 and m=3. The authors attribute this result to r·SD being close to the precision of the data. Still, this study demonstrates that relative consistency is at least subject to some technical conditions.

In [8], it is found that relative consistency is not preserved in very short (N=50) sinusoidal signals for sample entropy, while it is for the Fuzzy Entropy, a parameter that is introduced in that paper. While analyzing a similar set of measures, i.e., Approximate Entropy, Sample Entropy, and Fuzzy Measure Entropy, Zhao et al. [9] find that sample entropy does not behave consistently in distinguishing between normal sinus rhythm and congestive heart failure groups, which may be indicative of a lack of relative consistency. This paper is not entirely conclusive in this respect as it uses a segmented approach, and thus it is quite dissimilar to our study as well as the above-mentioned papers, but they do find that Fuzzy Measure Entropy is, as expected, totally consistent in this respect.

In this paper, we concentrate on the process which was used to demonstrate and study the properties of Approximate Entropy and SampEn, i.e., the MIX process. We contrast the results obtained for this process with a closely related process and show that their properties with respect to relative consistency are widely different. The considerations in this paper are limited to Sample Entropy because the fact that Approximate Entropy is not relatively consistent with respect to the MIX process has already been shown in [4].

### 1.1. Sample Entropy

Given a time series

(1) $$U_{i}=\left\{u\left(1\right),u\left(2\right),\ldots ,u\left(N\right)\right\},$$

where *N* is the number of data points, let us build an auxiliary object

(2) $$V_{i}^{m,\tau }=\left\{\vec{v}\left(1\right),\vec{v}\left(2\right),\ldots ,\vec{v}\left(N-\left(m-1\right)\tau \right)\right\},$$

which is a set of vectors in an *m*-dimensional *embedding space* [10,11], Vectors ${\vec{v}}_{m,\tau }\left(i\right)=\left[u\left(i\right),u\left(i+\tau \right),u\left(i+2\tau \right),\ldots ,u\left(i+\left(m-1\right)\tau \right)\right]$, i.e., the ${\vec{v}}_{m,\tau }\left(i\right)$ consist of *m* ordered points, beginning at position *i*. The parameter τ is known as time lag. Therefore, we have $L_{m}=N-\left(m-1\right)\tau$ vectors $V_{i}^{m,\tau }$ for a fixed τ—these are often called templates.

Let us define the so called correlation sum:

(3) $$C^{m}\left(r\right)=L_{m}^{-1}\sum_{i=1}^{L_{m}}C_{i}^{m}\left(r\right).$$

$C_{i}^{m}$ are defined as

(4) $$C_{i}^{m}\left(r\right)=\left\{\begin{array}{cc} {\left(L_{m}-1\right)}^{-1}\sum_{j=1,j\neq i}^{L_{m}}\Theta (r-|{\vec{v}}_{m}\left(i\right)-{\vec{v}}_{m}\left(j\right)\left|\right) & \mathrm{if}\;i\leq L_{m}\\ 0 & \mathrm{if}\;i>L_{m} \end{array}\right.,$$

Θ is called the *Heaviside* function

(5) $$\Theta \left(x\right)=\left\{\begin{array}{cc} 1 & \;\mathrm{if}\;x\geq 0\\ 0 & \;\mathrm{if}\;x<0 \end{array}\right..$$

where *r* is called the radius of comparison [12], which is used to check the similarity of two vectors by checking their distance with respect to a norm. The distance between two vectors in can be defined in many ways, but the following maximum coordinate distance definition seems to have the best mathematical properties [13]:

(6) $$|{\vec{v}}_{m}\left(i\right)-{\vec{v}}_{m}\left(j\right)|=\max_{k=1,2,\ldots ,m}(|u\left(i+\left(k-1\right)\tau \right)-u\left(j+\left(k-1\right)\tau \right)|).$$

SampEn (just like Approximate Entropy) is an attempt to build an estimator the Eckmann and Ruelle [14] entropy:

(7) $$ER=\lim_{r\to 0}\lim_{m\to \infty }\lim_{N\to \infty }\left[\Phi^{m}\left(r\right)-\Phi^{m+1}\left(r\right)\right],$$

where *N*, *r*, and *m* have the same definition as before. This expression involves limits, so it cannot be directly applied to a realistic, measured time series. In order to make this possible, this definition was rewritten by Richman and Moorman [4] to the following form for a finite time series:

(8) $$\Phi^{m}\left(r\right)={\left(N-m+1\right)}^{-1}\sum_{i=1}^{N-m+1}\log B_{i}^{m}\left(r\right),$$

$B_{i}^{m}$ has the same definition as $C_{i}^{m}$, with the only difference that self matches are included in $B_{i}^{m}$ and excluded from $C_{i}^{m}$. Richman and Moorman propose using the following, closely related quantity as complexity measure instead of the Eckmann–Ruelle entropy

(9) $$SampEn\left(m,r\right)=\lim_{N\to \infty }\left[-\ln\frac{C^{m+1}\left(r\right)}{C^{m}\left(r\right)}\right],$$

which, for a finite time series, can be estimated by

(10) $$SampEn\left(m,r,N\right)=-\ln\frac{C^{m+1}\left(r\right)}{C^{m}\left(r\right)}.$$

A detailed look into the constitutive elements of these formulas lead to the conclusion that Sample Entropy is the negative logarithm of the conditional probability that two sequences, which are within the *r* radius of tolerance of one another for *m* points, remain at the same radius of tolerance for m+1st point. A more detailed treatment of SampEn may be found in [4].

### 1.2. Relative Consistency

The notion of relative consistency was introduced by Pincus in [1,3], and this property follows from the properties of the Kolmogorov–Smirnov (KS) entropy. Rewritten in terms of SampEn, we have the following property: for deterministic dynamical processes *A, B*, we should have that, from KSentropy(A)<KSentropy(B), it follows that SampEn(m,r)(A)<SampEn(m,r)(B) and, conversely, for a wide range of *m* and *r*. This entails that, if $SampEn\left(m_{1},r_{1}\right)\left(A\right)<SampEn\left(m_{1},r_{1}\right)\left(B\right)$, then $SampEn\left(m_{2},r_{2}\right)\left(A\right)<SampEn\left(m_{2},r_{2}\right)\left(B\right)$ and vice versa. In other words, if SampEn for one process is lower than that for another process for a set of parameters $\left(m_{1},r_{1}\right)$, then this holds true for any other set $\left(m_{2},r_{2}\right)$ [4]. It should be stated clearly that this is an expectation and a desirable property of SampEn, rather than a mathematically proven property. If this holds true, then we are able to compare two processes at a single point $\left(m_{1},r_{1}\right)$ and draw conclusions for all points. This is what is actually happens in applications.

### 1.3. The MIX and MIXTURE Processes

Let us now define the two processes which will be used to test the relative consistency of SampEn under different conditions.

#### 1.3.1. MIX(p) Process

Let 0≤p≤1 be discrete probability. Let us define three time series [4]:

(11) $$X_{j}=\sqrt{2}\sin\left(j\right),$$

In the above, we do not use the frequency modifying factor $\frac{2\pi }{12}$ since our sampling is quite dense, as will become apparent in the Data Analysis section.

(12) $$Y_{j}=U\left(-\sqrt{3},\sqrt{3}\right),$$

i.e., uniform independent, identically distributed random variable, and

(13) $$Z_{j}=B\left(1,p\right),$$

i.e., a Bernoulli random variable with probability of success equal to *p*. We can now define the MIX(p) process as

(14) $$MIX\left(p\right)=\left(1-Z_{j}\right)X_{j}+Z_{j}Y_{j}.$$

#### 1.3.2. MIXTURE(λ) Process

This is a very simple process which is composed of a sum of two processes: a deterministic and stochastic process, the second of which is controlled by a tuning parameter λ. Let us define

(15) $$X_{j}=\sqrt{2}\sin\left(j\right),$$

(16) $$Z_{j}=U\left(0,1\right),$$

and the final process

(17) $$MIXTURE\left(\lambda \right)=X_{j}+\lambda \left(Z_{j}-0.5\right).$$

**Figure 1:**
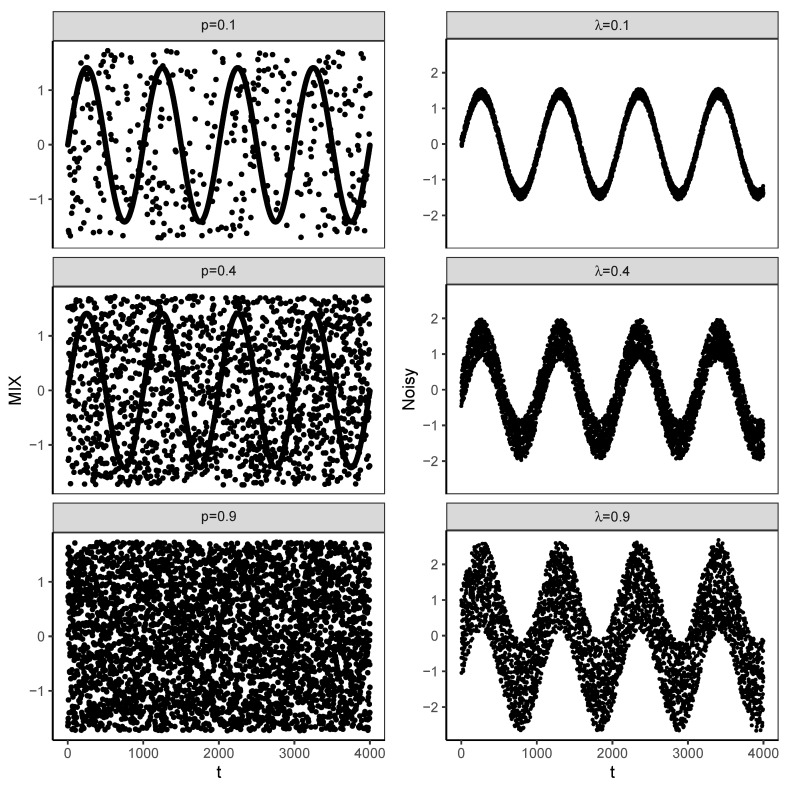
A few examples of MIX (left panel) and MIXTURE (right panel) processes. The level of distortion of the underlying deterministic signal increases from top (0.1) panels to the bottom ones (0.9).

It can be seen that the λ parameter controls the amplitude of the added noise. Figure 1 presents a few examples of of the above processes.

It should also be noted that, in spite of their apparent similarity, these two processes are very different. In the first of them, we tune how much randomness is in the signal, i.e., we control how many samples, on average, come from the random process. In the other process, we control how large the random effect is, i.e., what contribution *each* point gets from the random process. It may be argued that, in the MIX(p) process, the parameter (*p*) controls the amount of the random component in the deterministic signal, while the variance of a single random insertion remains the same, whereas in the MIXTURE(λ) process the amount of the random component is constant (maximum), and the parameter (λ) controls the variance of the random addition. This is interesting since it has been argued that the amount of variance in the analyzed time series affects the results of entropy calculations [15,16].

### 1.4. The NCM Algorithm

The NCM algorithm is the fastest algorithm for calculating correlation sums and SampEn which at the same time uses the whole time series, without any sub-sampling or simplifications.

This algorithm is of the *look-up* table type and uses many tricks limiting the number of operations as compared to the brute-force approach. The central objects of NCM are triangular matrices $\hat{N}$ whose elements are defined in the following way:

(18) $$n_{ij}=\left|\right|u_{i}-u_{i+(j+1)\cdot \tau }\left|\right|,$$

where *u* are the elements of the *U* time series and τ is the time lag. For a time series with *N* points, the dimensionality of this matrix is N−τ−1×(N−1)/τ−1. It is quite obvious that for any realistic time series this amounts to a very large matrix. The first operation-reducing technique is limiting calculation to sub-blocks. Using the symmetry of the matrix, elements with indices not meeting the condition i+(j+1)·τ≤N−τ−1 are set to zero, thus halving the number of summations in the correlation sum. Another result of this approach is removing redundant operations in calculating $C^{m}$ by reducing the number of operations for maximum norm from $m^{2}$ to *m*. The last operation is searching for the first occurrence of 0 from an arithmetic operation instead of a loop, with

(19) $$\left(n_{i,j}^{m}-b\right)/a,\;a=-\frac{r_{max}-r_{min}}{n-1},\;b=r_{max}.$$

Other, more standard optimization and algorithmic techniques as well as hardware scaling can be used to further improve the performance of the procedure. The full description of the algorithm may be found in [5] and Python code on the GPL license may be downloaded from https://github.com/sebzur/NCM-algorithm.

## 2. Materials and Methods

All the signals were processed with the use of the Python programming language. The correlation sums were calculated with the use of the NCM algorithm using the software available at https://github.com/sebzur/NCM-algorithm. The results were analyzed with the R programming language and statistical system.

### 2.1. The MIX Process

One hundred signals were generated with four thousand samples, and four periods were generated at the frequency of 5 Hz. The tuning parameter *p* for these signals changed from 0 to 1. For each of the signals, the correlation sums $C_{m}$ and $C_{m+1}$ were calculated for m=2 (compare Equation (9)) and SampEn was calculated using these results. The SampEn profiles were drawn for values of threshold *r* spanning the segment (0·SD,4·SD). As a result, we obtained 1100 plots for the different levels of the tuning parameter. We searched for crossing profiles within the plots by subtracting the profiles and counting how many times the difference changed sign.

### 2.2. The MIXTURE Process

One hundred signals were generated according to the definition from formula (17). The parameters assumed were the same as for the MIX process, and the λ tuning parameter also changed in the range λ∈(0,1). We calculated correlation sums and SampEn profiles for the same sets of parameters as in the MIX process case. As before, we got 1100 plots and we checked for crossing profiles.

## 3. Results

### 3.1. The MIX Process

**Figure 2:**
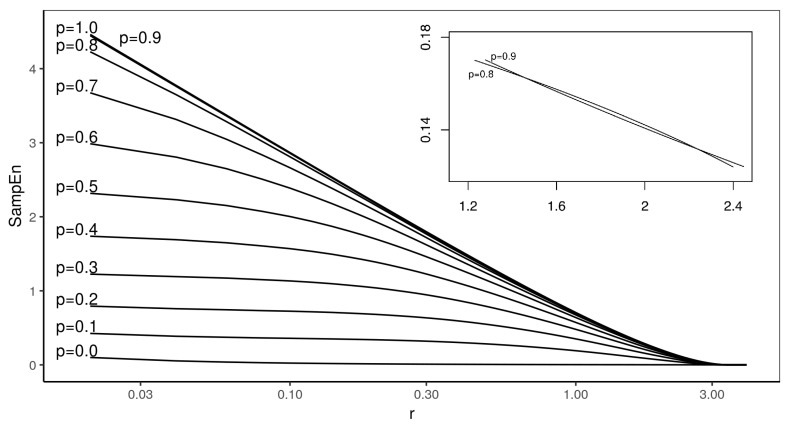
In this figure, entropy profiles of the MIX process calculated for m=2 embedding are presented. Each line corresponds to different *p*—starting from 0.1 with step 0.1. The inset presents the close up of crosses between entropy profiles for p=0.8 and p=0.9 in linear scale.

Figure 2 presents the entropy profiles for m=2.

We have observed that there are crossings in the entropy profiles. All the crosses in the present study behave in the same way: the lines always cross at two points, which means that there is a finite region for which the order of the lines reverses. The crosses have been summarized in Table 1 below. For each line, it contains two values of *r* at which the lines cross, and the SampEn value at the crossing point. It is interesting to notice that many entropy profiles cross with the maximum randomness MIX(1) process, and there is also one more case which does not involve this process, namely MIX(0.8) and MIX(0.9).

### 3.2. The MIXTURE Process

**Figure 3:**
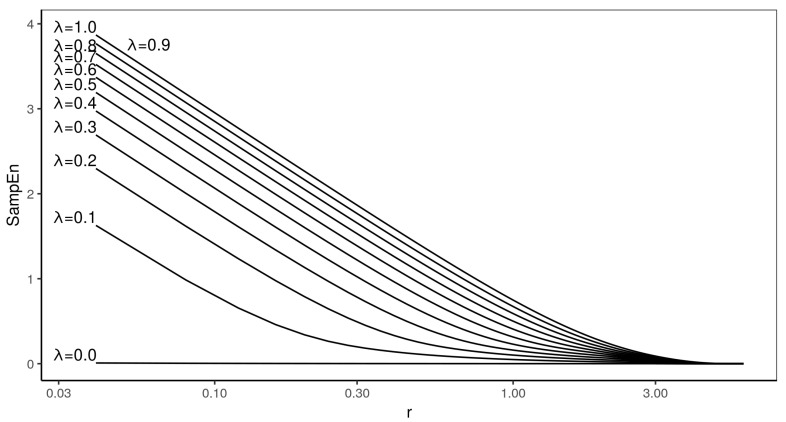
In this figure entropy profiles of MIXTURE process calculated for m=2 embedding are presented. Each line corresponds to different λ—starting from 0.1 with step 0.1. The lowest SampEn value corresponds to λ=0.1.

Figure 3 presents the entropy profiles for m=2. There are no crossings in this type of process, irrespective of the value of λ.

## 4. Discussion

In the present paper, we have studied the relative consistency property of the Sample Entropy parameter. We have experimentally studied two synthetic time series—the MIX(p) and MIXTURE(λ) processes. Both of these processes have one tuning parameter; however, in the MIX(p) process, the amount of randomness is controlled, and, in the MIXTURE(λ) process, the size of the random effect is controlled. It turns out that relative consistency is not preserved for the former, while it is preserved in the latter.

The nature of Sample Entropy profiles crossing is different from that found in [6], or the behavior observed in Approximate Entropy [2], which is a flip behavior. In our analysis, the entropy profiles for MIX(p) always cross twice. Of course, the reason could be the lack of possibility to observe the other cross in the above cited studies.

It is also interesting to notice that, though the amount of variance in a time series has been reported to influence the results of entropy calculations [15,16], this does not seem to be the case for the relative consistency of MIXTURE(λ), which is preserved for all values or λ.

A question may arise whether the values of *r* at which the crosses were found are important for practical applications. In our opinion, it is impossible to relate the value of *r* for the MIX process with a value of *r* from, say, an RR intervals time series. These are two completely different processes and the values cannot be compared. The MIXTURE process is in fact a good example here—there are no crosses for this process, so no crossing value of *r* in MIX corresponds to any *r* value in the MIXTURE process.

We believe that finding a process which is clearly relatively consistent for all values of the parameters, as opposed to a process which is mostly relatively consistent, is one of the most important results of this paper. This finding may have deep consequences.

In applications to medicine and economy relative consistency is assumed anywhere where two signals are compared and conclusions are drawn on the basis of a single set of parameters (m,r). It is absolutely necessary to study real signals, e.g., time series of RR intervals, using the methodology we have presented in this paper or a similar one. If the studied real signals behave more like the MIXTURE(λ) process, then we can continue assuming relative consistency, if they behave more like the MIX(p) processes, the approach to comparing signals may need to be modified.

**Figure 4:**
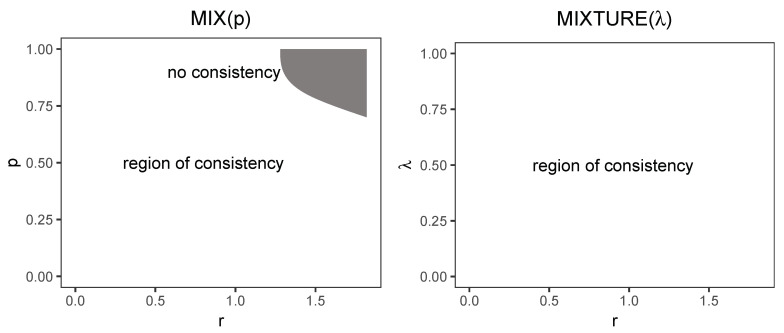
Relative consistency region for the MIX(p) and MIXTURE(λ) processes for p∈(0,1) and r∈(0,1.82). The region for MIX(p) has been smoothed by natural splines to interpolate the values between measured points.

Looking at the results obtained in this manuscript as well as the cited papers, we can notice that various processes have various *regions of relative consistency*, i.e., a region in a multidimensional space within which the process is relatively consistent. For the MIX(p) region, we can see that process is relatively consistent for a certain region in the (r,p) space, and, for the MIXTURE(λ) relative consistency, holds for the entire studied region in the same space. These regions have been demonstrated in Figure 4.

We suggest that such regions be found for real-life processes, and, from the data gleaned from other studies, we can suspect that they will be different for different processes and for different spaces. For example, the results presented in [6,7] suggest that for the gait signal there is at least one region where the signal is not relatively consistent in the (r,f) space, where *f* is sampling frequency, and the point (0.2,148Hz) belongs to this non-consistency region (for a study on the influence of sampling frequency on SampEn see also [17]). The same two studies by Yentes et.al. suggest that other spaces of interest could be (r,N), where *N* is the length of the time series, and (N,f) or even some multidimensional spaces involving these variables can be studied in this way.

The RR intervals time series seems to be a good candidate for such a study because of its widespread use, equipment availability, and the fact that many researchers are already very familiar with this time series. Such a study would require a group of recordings from similar subjects, taken with the same equipment, in stationary conditions. They would also need to be long enough to let the underlying process assume as many intermediate stages as possible. In our opinion, the main obstacle in studying longer time series is the computational burden of calculating entropy profiles. The method described in this paper as well as the open source software we make available makes this possible.

We believe that the answer to the question of the regions in which relative consistency holds in various types of signals is one of the most important problems for the applicability of SampEn because, in order to be able to safely compare the values of SampEn at selected points, we need to make sure that the comparisons take place within a region of relative consistency.

## Figures and Tables

**Table 1:** Crosses between entropy profiles for the MIX(p) process.

Crossing Lines	$r_{1}$	SampEn	$r_{2}$	SampEn
p0.7, p1.0	1.82	0.2565933	2.14	0.1591130
p0.8, p0.9	1.50	0.3900531	2.30	0.1206111
p0.8, p1.0	1.40	0.4402257	2.26	0.1298239
p0.9, p1.0	1.30	0.4964603	2.24	0.1344648
